# Perils and promise providing information on sexual and reproductive health via the Nurse Nisa WhatsApp chatbot in the Democratic Republic of the Congo

**DOI:** 10.1080/26410397.2023.2235796

**Published:** 2023-08-07

**Authors:** Emily McMahon, Tamara Fetters, Nadia Lobo Jive, Mike Mpoyi

**Affiliations:** a Senior Advisor, Ipas, Chapel Hill, NC, USA.; b Senior Research Scientist, Ipas, Chapel Hill, NC, USA; c Research Associate, Ipas, Kinshasa, Democratic Republic of Congo; d Manager, Ipas, Kinshasa, Democratic Republic of Congo

**Keywords:** abortion, contraception, sexual and gender-based violence, chatbot, knowledge platform, lessons learned, scale-up, social media, censorship, Democratic Republic of Congo

## Introduction

Having access to accurate and reliable sexual and reproductive health (SRH) information is a fundamental human right and a moral imperative in low-income countries with under-resourced health systems. A person not in control of their own fertility faces many challenges and barriers to community participation and overall quality of life. Providing direct access to accurate and reliable SRH information can make it possible for a person to manage their health, overcome barriers, and transform society as a whole.^1^ Denial of that information represents a grave injustice and denies people their right to make autonomous decisions regarding their health.^2,3^ Providing evidence-based information to allow people to control if, when, and how to have children is a widely shared and agreed upon goal of global public health, essential for achieving gender equality as set out in the Sustainable Development Goals.^4^ Providing this information in low-income countries with under-resourced health systems is a moral imperative.

Accurate and trustworthy information is particularly imperative to ensure pregnant people can access safe and timely abortion care, including medical abortion that is self-managed. Self-managed abortion is safe and effective when performed according to WHO guidelines,^5,6^ and there is a growing history of digital, mobile, and other resources being used to share information among under-served groups about self-managed abortion.^7–12^ The tools and information to control one’s own fertility exist; the challenge of our time is how best to facilitate access to them in a time when misinformation is rampant.

The Democratic Republic of the Congo (DRC) continues to face significant challenges to the delivery of reproductive health services due to conflict, lack of health infrastructure, a limited resource base for health care spending, and multiple pandemics.^13,14^ Early marriage is common in the DRC, and nearly half of Congolese women become pregnant before they turn 20.^15^ Only 21% of unmarried, sexually active women and 8% of married women are using modern contraception and over one-quarter of married women have an unmet need for family planning.^15^ The DRC also ranks 179th in the world (out of 191 countries) in the gender equality index, affecting every aspect of a woman’s life and livelihood, including her reproductive decision-making.^16^

In 2014 it was estimated that unsafe abortion in the DRC caused nearly 9% of the country’s maternal deaths,^17^ in a country with the 23rd highest maternal mortality ratio in the world,^18^ resulting in thousands of preventable deaths and countless morbidities each year. The 2018 reform of abortion policies in the DRC expanded indications under which abortion is permitted from criminalisation and severely restricted access to the authorisation of medical abortion in cases of sexual assault, rape, incest, and where the continued pregnancy endangers the mental and physical health of the mother or fetus.^19^ These changes are in line with the country’s commitment to the ratification of the Protocol to the African Charter on Human and Peoples’ Rights on the Rights of Women in Africa (the Maputo Protocol).^19^ However, implementation has been slow, and many women still lack meaningful access to abortion care due to gaps in the health system, lack of commodities, lack of knowledge about the legal status of abortion, knowledge about where to obtain safe services, and the stigmatisation of abortion.^13,14^

The COVID-19 pandemic and measures taken to mitigate its spread presented new challenges in the delivery of and access to SRH care globally. Limited mobility due to stay-at-home orders and efforts to control the spread of the virus impeded access to clinical settings. Millions of women and girls faced contraceptive interruption, unintended pregnancy, and unsafe abortion as healthcare workers struggled to meet increasing demands on their time.^20,21,22^ Yet disruptions in access to SRH services were not limited to repercussions of the COVID-19 pandemic. The DRC has been subject to ongoing crises for decades: from natural disasters to civil wars resulting in the internal displacement of over five million Congolese, and becoming the temporary home to over a half million Africans fleeing neighbouring crises.^23^

In this complex context, a digital solution emerged as the most feasible and scalable solution to the widespread information needs. Internet penetration in the DRC stood at 19% of the population in January 2020, but had increased by nine million (+122%) between 2019 and 2020.^24^ Nearly 40% of the population had mobile connections.^24^ The rapidly growing internet and cellular penetration resulted in an increase of 680,000 (+28%) social media users between 2019 and January 2020.^24^ In early 2023, Facebook had 4.7 million users in the DRC.^24^ WhatsApp was selected as the ideal information conduit because it is available on both feature and smartphones and is inexpensive, using low amounts of data compared to other internet-based options. All aspects of connectivity and social media use are growing rapidly in the DRC.

The WhatsApp-based Nurse Nisa chatbot provides confidential SRH information on the stigmatised topics of abortion, contraception, emergency contraception, and gender-based violence. Providing this information directly to people, including young people, enables them to make reproductive choices that can help them remain in school, continue to provide for their families, and make autonomous decisions about their health and fertility. The information shared is simple yet evidence-based, providing an opportunity to explore information on controversial or stigmatised SRH issues in a confidential way.

This article describes lessons and insights from a three-year process of developing, prototyping, and scaling up the Nurse Nisa chatbot in the DRC by two international non-governmental partners, Ipas, focused on reproductive justice and Dimagi, on social enterprise. The lessons provided can help others working to improve information on sensitive topics in innovative ways.

## Creating and piloting the Nurse Nisa chatbot

Ipas is a global non-governmental reproductive justice organisation dedicated to ending preventable deaths and disabilities from unsafe abortion and prevention of unintended pregnancies, with a national programme in the DRC. Following an approach in early 2020 by the social enterprise technology organisation, Dimagi, whose objective is to help scale-up digital technology solutions in global development, the two partners joined forces to develop and prototype a digital tool to improve comprehensive evidence-based information on sexual and reproductive health to online users. Together we created a direct-to-client digital solution, the Nurse Nisa chatbot. Initially designed to improve access to accurate information about contraception and self-managed abortion for people isolated and afraid to seek facility-based care during the COVID19 pandemic, Nurse Nisa was later expanded to include information on identifying gender-based violence and coercion in your relationships. Nurse Nisa runs on the Turn.io platform, a low-cost solution hosting option with monthly and annual subscription pricing which operates using WhatsApp. The low code environment developed by Turn.io enabled us to build and iterate quickly based on user feedback and chatbot analytics.

## Content development and initial prototyping

Building on Dimagi’s experience creating technology solutions for frontline health workers and users in low- and middle-income countries, we built a chatbot prototype that provided accurate uncensored information, including controversial topics like religious objection, myths about abortion, emergency contraception, and complete instructions for self-managed abortion. Relying on Ipas’s global network of technical experts, prototyped content and navigation were created simultaneously in English, Swahili, Hindi, and French, and pilot-tested in three countries in the Ipas network with the strongest interest, cellular penetration, and need – the DRC, Kenya, and India – in the last months of 2020 and early 2021. The different country programmes approached prototyping and dissemination differently – relying on organised user-centred design groups, testing and monitoring analytics, or relying on feedback from peer and community educators – and thereby generated customised chatbots and different engagement results. Relying on their networks of community partners, the highest engagement was seen in India, where peer and community health workers used peer-to-peer introductions to the chatbot to introduce content with users in facilitated conversations. After prototyping experiences, each of the three pilot countries chose to adapt their digital work in different directions. The India programme determined that more investment in a chatbot utilising artificial intelligence would be more useful for their work. The Kenya programme continues to field Nurse Nisa as a companion to its website and online platform for adolescents, Nimechanuka. DRC collaborators determined that their interest was in expanding the SRH content to new and important issues, such as gender-based violence and coercion, and increasing the user base with an extensive social media presence.

## Scaling up to address other SRH topics and increase users

In April 2021, after piloting and prototyping in the DRC, we decided to reassess the chatbot and explore related SRH issues and pressing needs in the DRC with a continued focus on improving knowledge related to women’s bodily autonomy. A chatbot remained the ideal solution for improving knowledge because it allowed users to access stigmatised and sensitive content at any time outside of the health system, especially as the COVID-19 pandemic was still disrupting in-person visits to health facilities. Sexual violence emerged as a dire and under-discussed issue among stakeholders. Gender inequality in the DRC, exacerbated by poverty and instability, drives and normalises violence against women and girls; nearly 1150 women are raped every day and approximately 35% of women experience sexual violence with intimate partners (IPV) in their lifetimes.^25^ Addressing violence and coercion allowed for broader contextualised information that promoted our rights-based framing of gender equality, bodily autonomy, and agency over one’s own reproductive choices. New content was created in the format of six short stories written to describe different types of violence and reproductive coercion, including economic abuse, emotional abuse, psychological abuse, sexual abuse, physical abuse, and coercion. Alongside the stories is a connection to a government-sponsored hotline number that provides live support to women in crisis. With the three main pillars of content ready – abortion, contraception, and gender-based violence – Nurse Nisa was ready for scale-up.

**Figure 1.**
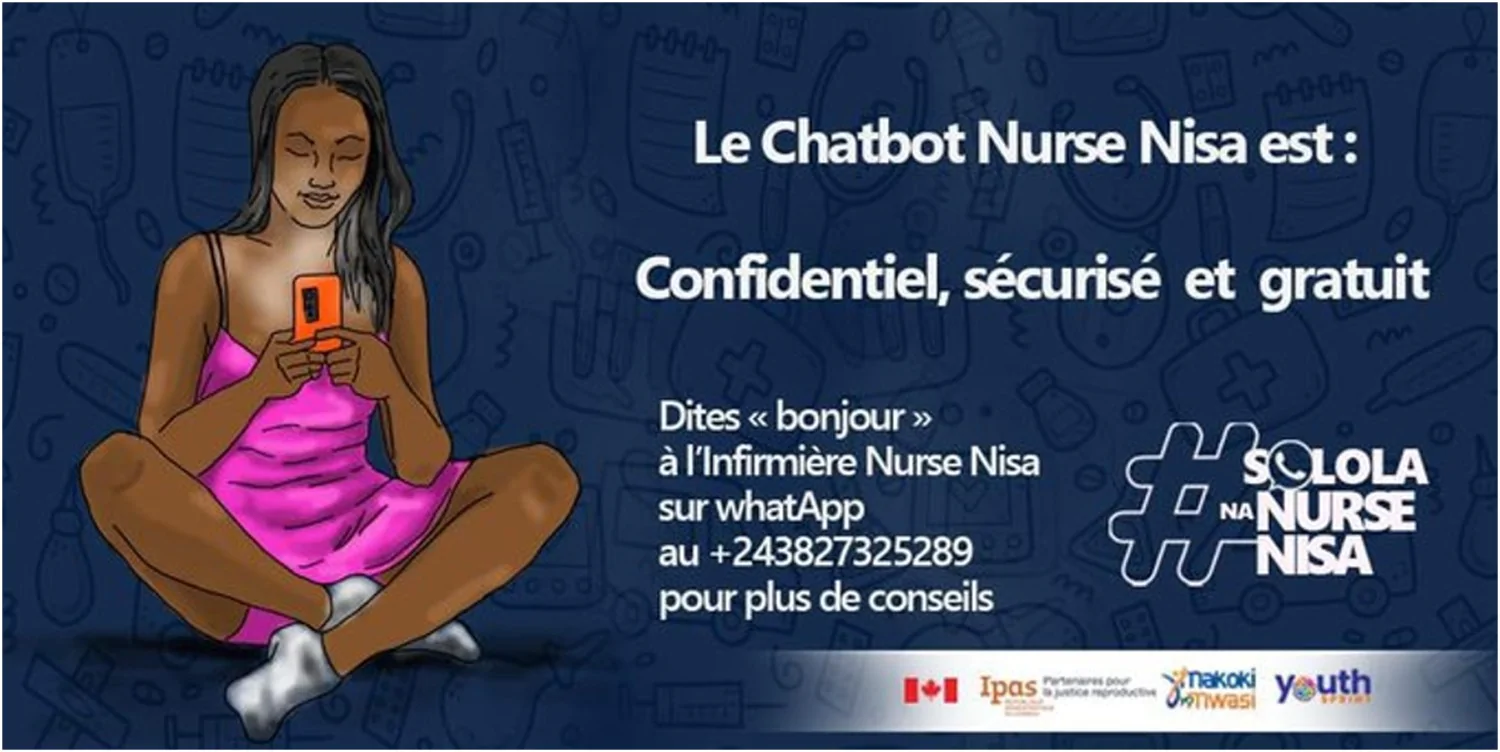
Example of a social media campaign product used with a local youth organisation.

Scaling up Nurse Nisa was reliant on two main factors, increasing users and improving engagement. Chatbots do not grow themselves; the service must be in front of people either through digital platforms, like social or mass media, or shared during meetings, trainings, or integration with campaigns of local partners willing to include abortion content. Increasing users was primarily approached through online advertising and local promotion. An introduction to Nurse Nisa was integrated into all Ipas programmatic work with the QR code included on business cards, on posters, presentation materials, and in all meetings or events. Additionally, six social media campaigns were launched in partnership with Youth Sprint, a local youth-focused organisation. Social media campaigns (Figure 1) generated intense engagement online and always created a spike in chatbot users. Social media were used to drive users to the bot, while community-driven campaigns helped to sustain engagement incorporating live music, engaging speakers, and Wi-Fi hot-spots. Youth conferences were organised in schools to tackle misinformation and myths about abortion and share the chatbot with young people so they could confidentially access more complete information. Some community campaigns and events targeted hard-to-reach population groups of refugees and displaced people such as those in Goma, Bukavu, and Haut Katanga, relying on media opportunities, existing festivals, and events to introduce Nurse Nisa. For example, in Goma, a conflict-affected region, a peace-building campaign called the *Amani festival*, encouraging cooperation and peace between tribes, was a popular forum for in-person promoters of the chatbot highlighting sexual and gender-based violence (SGBV) content at the anti-violence event.

## Analytics and insights

We sought an expert partner, Tangible AI, to assist in the development of an analytics dashboard to help us better comprehend and use data generated by Nurse Nisa. Chatbot analytics fall in three main categories: users, messages, and content. Since April 2021, the Nurse Nisa chatbot attracted over 7000 unique users (individuals that viewed one or more messages) in the DRC; throughout the pilot and scale-up phases from late 2020 to 2023, 1500 Nurse Nisa users returned to view content two or more times.

**Figure 2.**
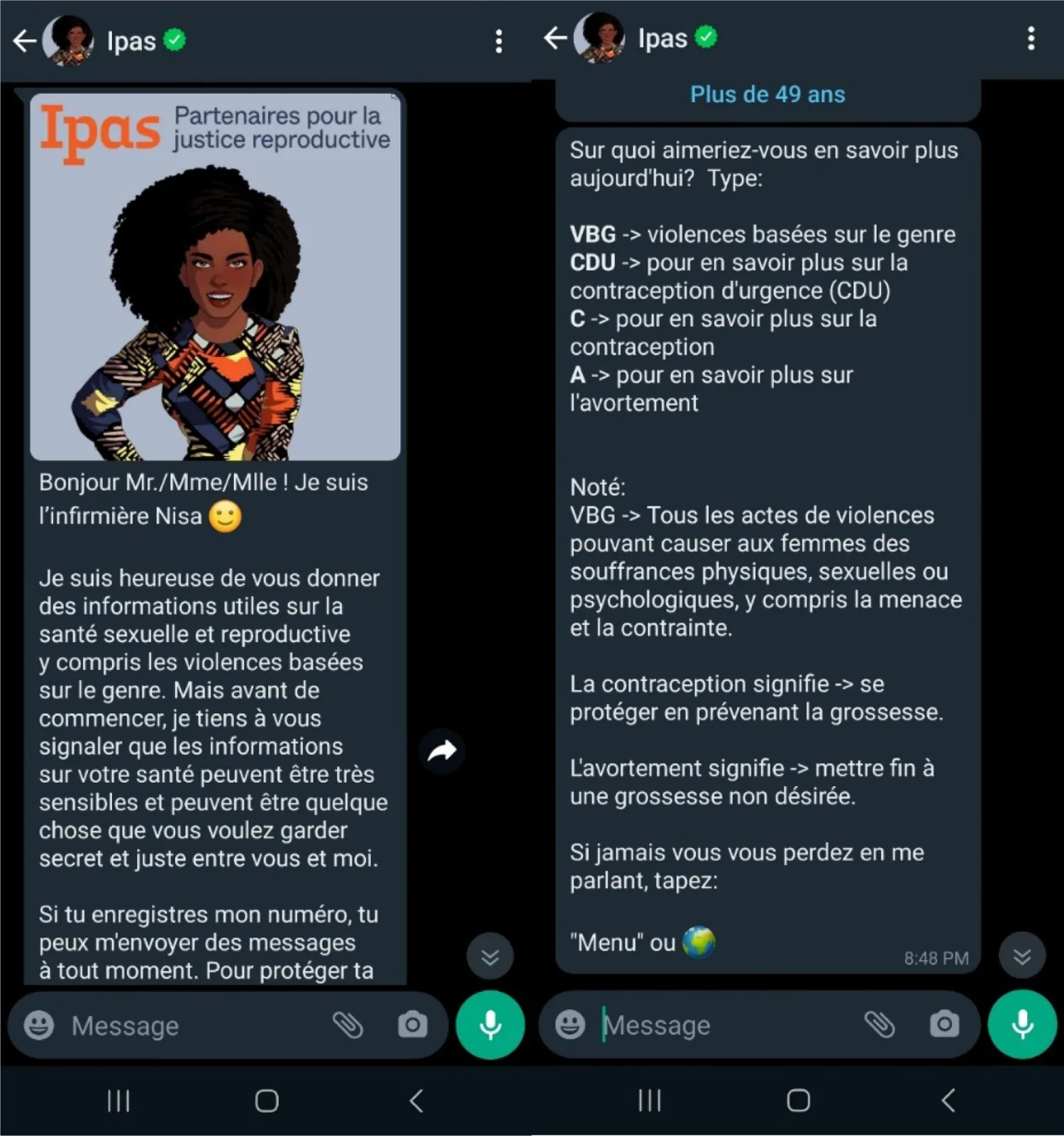
Screenshots of the Nurse Nisa chatbot welcome messages and main menu.

The functional design of the chatbot was created to be user-driven with all content developed by technical experts and contextualised for users in their own countries during the pilot phase. The chatbot does not rely on artificial intelligence or large language models for information; as such, the content is limited to the pre-defined domains of abortion, contraception, and gender-based violence. Users begin the conversation by messaging the chatbot in WhatsApp and the chatbot replies with a welcome message and a question about whether they wish to receive messages. Only users that consent to continue the interaction receive instructions on how to get more information (Figure 2). For example, a user will enter acronyms/abbreviations related to the menu or sub-menus, entering “VBG” to access the SGBV content, allowing for a personalised and self-guided experience. The menu uses short text prompts that need to be typed, rather than emojis or numbers, which resulted in more confusion and shorter use patterns on Nurse Nisa during the pilot. However, some users cannot or do not always follow navigation instructions resulting in unprompted text and questions. This anonymised information is periodically reviewed to improve navigation and better understand user intentions and problems. A set of common questions emerged from these reviews which led to the development of a frequently asked questions (FAQ) list. The FAQs answer questions such as how to terminate a pregnancy, how to avoid becoming pregnant, and how to use emergency contraception. While most answers are already provided by the chatbot, new shortcuts, and reinforced messages help low-literacy users and those who do not fully read the information in the main menus. We also discovered that users wanted to thank the chatbot and for this we developed a “you are welcome” message. Future changes will continue to focus on message clarity and natural language processing.

Almost no private or demographic data are collected in this chatbot, although some information is required for user registration on the WhatsApp platform. The Nurse Nisa chatbot has only two optional questions for users, gender and age. Requesting basic demographic information, like age and gender, allows implementors to better understand their user groups but needs to be balanced with concerns about user privacy and fatigue. Over one-third (36%) of users have supplied this information, allowing for some evaluation of engagement across these two dimensions. Of those who indicate their sex and age, 59% are men, 41% are women, 87% are between the ages of 20 and 49, and 11% are under 20. The higher percentage of men is not surprising, given the known gender gap in mobile phone use globally and in the DRC.^26^

The second key chatbot analytic is message volume: if volume increases, it indicates that users are exploring more widely and engaging with more content. The bot has sent over 165,000 content messages to interested users over the lifetime from pilot to scale-up, with each user viewing an average of 24 messages. When analysing message volume by the three main content categories, the newer SGBV content was found to be most engaging, accounting for 44.6% of message volume. This means that users were requesting and receiving several messages across the SGBV content. Abortion content was second in popularity, occupying 36.5% of message volume. Contraception occupies the least amount of user time, 18.9% of message volume.

Analysing content is different from message volume because it focuses on the impact of one piece of information. Over four in ten (44%) of users received educational content from the bot, by proceeding past the welcome, opt-in informed consent, and moving on to educational content accessed through the menus. Looking across all content messages, the emergency contraception (EC) message, that has been included since the inception of Nurse Nisa, has the highest number of unique users, meaning it was the most popular individual piece of content across the bot. Additional pieces of content with a high number of users include the definition of a “healthy and loving relationship”, general abortion information, help with gender-based violence in your life, sexual violence, and contraception for youth. Many similarities exist between the content interests of men and women. Emergency contraception and contraception for young people are similarly the top topics in those two areas across genders. However, in the SGBV content there are gendered differences in data exploration. The most popular SGBV content for women is healthy relationships and the most popular content for men sexual violence.

## Advertising and censorship

Maintaining interest and enthusiasm in a chatbot requires communications and social media expertise to build and sustain the user base. Our experience indicates that engagement drops dramatically when social media advertising ends. While some users began exploring Nurse Nisa through natural or “organic” posts of followed organisations or friends, these were supplemented by paid social media influencers or pop-up advertising. Monitoring and tracking advertisement performance is an integral component of the digital space. Utilisation and continuous performance monitoring of a small number of analytics is essential to determine what resonates with your audience on different social media platforms.

Paid advertising (Figure 1) provides rapid increases in views and information necessary to improve content in this digital space where interfacing with users is impossible. However, Facebook’s combined automated and human review process uses a stringent advertising policy that limits, sometimes unknowingly, and censors information on SRH. Legitimate SRH information on abortion or contraception is often rejected for falling under the categories of Adult Content, Controversial Content, or Illegal or Adult Products or Services, leaving few options for ensuring users can make informed choices related to their needs.^27^ Images with SRH content are quickly rejected or removed, and most SRH content is restricted for users under 18, leaving few options for connecting adolescents and others in need of information on essential SRH care. The solution deployed to mitigate censorship included removing censored keywords like “contraception”, “pregnancy”, and “abortions”, that can flag a post for blocking, demonstrated in Figure 1. The main text of the ad (Figure 1) says “The Nurse Nisa chatbot is: confidential, secure and free” with the WhatsApp number on every post. Yet it is likely that many users who need Nurse Nisa don’t connect due to the nuances and censoring of these posts.

## Seeking more information on impact and users through embedded surveys

In 2022, an optional quiz was developed as a new way of engaging users and accessing knowledge. The quiz consists of eight questions, four on SGBV, two on abortion, and two on contraception. The questions and answers were written to reinforce messages about the safety and legality of abortion, contraception, and SGBV awareness by providing the correct answer and reinforcing information when an incorrect option is chosen. The quiz was finished by 1219 of the 1410 users that started the quiz, for an 86% completion rate. Currently, 77% of those that completed the quiz had four or more questions correct.

Surveys offer an opportunity to gain insight about impact, such as changes in knowledge, but also potentially questions on behaviours, such as calling a hotline or using abortion pills. The temporal measurement issues of absorbing information and acting upon it can be addressed with pre-programmed “push messages” that include opportunities to further engage with or answer questions in an opt-in survey by following up with consenting users after initial contact. In the case of Nurse Nisa, an invitation to take a quiz is sent to consenting users 23 hours after they opt-in to receive messages without charge to the user or developer. These types of “push messages” are an inexpensive way to reinforce behaviour change messages, collect impact data, or reengage users.

## Conclusions and recommendations

As the digital divide between countries and genders continues to narrow, even with their limitations, chatbots offer a low-cost opportunity for providing and maintaining high-quality evidence-based information with a personalised and private experience. As new chatbots are developed, many powered by artificial intelligence (AI), the world is most certainly facing continuous growth in the chat for impact space, including the sphere of SRH. It is likely that digital competition will increase and new ways of engaging and maintaining a user base will need to be invented. Even as chatbots become automated by AI there is a firm place for human efforts, especially in the realm of evidence generation, content technical review, product promotion, and quality assurance. It remains the job of SRH practitioners to ensure quality of content, protect data privacy, and monitor ethical considerations. In practice this work includes strong technical review of content, incorporation of informed consent to participate, and a need to safeguard participants, especially young users. Having the ability to scale-up and test life-changing messages in controlled and randomly assigned ways to improve their efficacy with simple analytics such as return visits, engagement time and content exploration is certainly appealing. Evidence on the efficacy of chatbots for knowledge and behaviour change is limited at present but chatbots and the evaluation of these tools are growing rapidly.^28^ True randomised controlled trials that measure the impact of digital and telecommunication tools – whether chatbots, websites, mobile applications, or hotlines – compared to one another and against human interaction, are essential to show value for money. Furthermore, advocacy is still needed to combat censorship from large media outlets to ensure these products are chosen by knowledgeable and informed users. Access to information and scientific advances is a human right, and chatbots offer a scalable and an innovative way of democratising SRH information around the globe.
